# Overcoming Barriers to Injectable Therapies: Development of the ORBIT Intervention Within a Behavioural Change Framework

**DOI:** 10.3389/fcdhc.2021.792634

**Published:** 2021-12-09

**Authors:** Karen McGuigan, Alyson Hill, Deirdre McCay, Maurice O’Kane, Vivien Coates

**Affiliations:** ^1^ School of Nursing and Midwifery, Queen’s University Belfast, Belfast, United Kingdom; ^2^ School of Biomedical Sciences (NICHE), Ulster University, Coleraine, United Kingdom; ^3^ Western Health & Social Care Trust, Londonderry, United Kingdom; ^4^ School of Nursing, Ulster University, Coleraine, United Kingdom

**Keywords:** type 2 diabetes, behaviour change, psychological, intervention, injectable therapies

## Abstract

It is estimated among individuals with type 2 diabetes (T2D) requiring injectable therapies to achieve optimal glycaemic control, one-third are reluctant to initiate therapies, with approximately 80% choosing to discontinue or interrupt injectable regimens soon after commencement. Initiation of injectables is a complex issue, with effectiveness of such treatments undermined by non-adherence or poor engagement. Poor engagement and adherence are attributed to psychological aspects such as individuals’ negative perceptions of injectables, depression, anxiety, feelings of shame, distress and perceived lack of control over their condition. The aim of this study was to describe the development of a structured diabetes intervention to address psychological barriers to injectable treatments among a cohort of those with T2D; conducted within a behavioural change framework. An evidence base was developed to inform on key psychological barriers to injectable therapies. A systematic review highlighted the need for theory-based, structured diabetes education focussed on associated psychological constructs to inform effective, patient-centric provisions to improve injectable initiation and persistence. Findings from the focus groups with individuals who had recently commenced injectable therapies, identified patient-centric barriers to initiation and persistence with injectables. Findings from the systematic review and focus groups were translated *via* Behavioural Change Wheel (BCW) framework to develop an intervention for people with T2D transitioning to injectable therapies: Overcoming and Removing Barriers to Injectable Treatment in T2D (ORBIT). This article describes how psychological barriers informed the intervention with these mapped onto relevant components, intervention functions and selected behaviour change techniques, and finally aligned with behaviour change techniques. This article outlines the systematic approach to intervention development within the BCW framework; guiding readers through the practical application of each stage. The use of the BCW framework has ensured the development of the intervention is theory driven, with the research able to be evaluated and validated through replication due to the clarity around processes and tasks completed at each stage.

## Introduction

The complexities of treatment regimens for those living with Type 2 Diabetes (T2D) can be problematic, contributing to issues with medication adherence among this population (1). This is particularly salient among those who require the use of injectable therapy i.e. GLP-1 receptor agonists or insulin, to achieve optimal glycaemic control (2). It is estimated 1 in 3 people are reluctant to begin prescribed injectable therapy, with approximately 80% believed to discontinue or interrupt injectable regimens quite quickly after commencement (3–5). Issues of non-adherence or poor engagement with injectables serves to undermine the effectiveness of these treatments. Poor injectable uptake and adherence have been attributed to *“significant barriers in the minds of patients”* [(6), s12]. Psychological aspects such as individuals’ perceptions of injectables, depression, anxiety, fear of injections, perceived pain, feelings of shame and failure can impact on engagement with therapies of this type (7–9). These psychological aspects are associated with poorer: clinical outcomes, initiation of injectable therapies, medication adherence and motivation which impact negatively on effective self-management behaviours (9–13).

Non-adherence to medication regimen, medical guidelines or treatment targets can be intentional or not, with people living with diabetes making conscious or sub-conscious determinations about the benefit of the treatment against the potential impact on daily functioning, wellbeing and quality of life (14). The literature supports the links between individuals’ perceptions of their condition and their motivation to adhere to prescribed treatments (15, 16). People with diabetes report concerns around treatment complexity, the restrictive nature of injectable regimens and the impact on day-to-day living (17). Poor knowledge about the use of injectables, particularly insulin, can affect confidence in their use (9). This, in turn, serves to increase the risk of associated complications, increase diabetes related distress, and adversely affect glycaemic control (9, 11).

As T2D is a condition that is primarily managed by the individual, efforts to address patient-related challenges of the condition have been recommended to improve self-management, medication adherence and outcomes (2, 18). However, despite clear recognition of the importance of behavioural change to ensure effective self-management of T2D; behavioural change techniques and the psychological aspects which affect behaviour change have been overlooked in the development of structured diabetes interventions (19). There has been significant underinvestment in interventions which target behaviour change with greater focus on the development of medications and devices to affect better outcomes (19). Overcoming the challenges presented by injectable therapy would be best served through an educational intervention (6, 9). However, the intervention must reflect best practice guidelines for structured diabetes education (20), respond to practical aspects of injectable use, and address psychological barriers to injectable uptake and maintenance (6, 9, 21).

When developing a new or novel intervention to change behaviour, few researchers provide a detailed description of the intervention development stages or processes, resulting in a lack of clarity for evaluation or replication of the intervention (22). Whilst researchers suggest a theory or framework underpinning intervention design, this is often either poorly described or applied (23). In other instances, intervention design is not guided by a theoretical framework, suggesting perhaps the existing options are not suited to the intervention aims (24). The Medical Research Council (MRC) has provided guidance on the use of theoretical frameworks in intervention design, emphasising their effectiveness in identifying mechanisms for behavioural change (25). Evidence-based interventions using appropriate theoretical frameworks are more likely to be successful in changing targeted behaviours (26).

Understanding and consequently attempting to change health behaviours is not a simple task. However, interventions aimed at affecting behaviour change are more likely to be effective if they are grounded in key psychological principles or theories of behaviour change (27). Given the demands self-management places on the individual living with T2D, it is important to recognise the behavioural adaptations required to ensure optimal glycaemic control for those on injectables. This requires adherence to their treatment plan, monitoring blood glucose levels, improving diet and physical activity levels, as well as attending regular healthcare appointments (14). Unsurprisingly, living with T2D has been described as a *“chronic stressor for patients and families, affecting various life domains”* (14,p.541). Accordingly, a behavioural change theory with a singular focus and little consideration for contextual factors may not be the best fit for an intervention to overcome barriers to injectable treatments for those with T2D. Roter et al. (28) were among some of the earlier researchers to assert that more comprehensive interventions with a combined focus would yield better outcomes, with single-focus interventions exhibiting less efficacy. They recommend interventions reflect psychological, behavioural and affective aspects to inprove effectiveness. Education provision for successful management of T2D using injectables cannot have a singular focus on injections or simple provision of information; instead requiring training on appropriate behavioural skills, coping strategies, individual practice, feedback and support (14, 29). Indeed, whilst interventions based on a single theory may be easier to evaluate, they do not provide a comprehensive assessment of a clearly operationalised behavioural change problem (23). A comprehensive theoretical framework for behavioural change is required.

The Behaviour Change Wheel (BCW) is a comprehensive theoretical model for behaviour change which was developed from a synthesis of 19 existing behavioural change frameworks, in essence ensuring a model that reflects their best practice from those (26, 30). Accordingly, the BCW overcomes many of the issues that have hampered other frameworks or theories of behavioural change (31). The BCW advocates a systematic approach for intervention design (32) offering a pragmatic, theoretical framework for health intervention development and evaluation that has been shown to successfully facilitate behavioural change (24).

The National Institute for Health and Care Excellence (33) guidelines for behavioural change advocate behaviours are a result of the interface between an individual’s capability and opportunity to perform that behaviour, and their motivation to do so. Reflecting this guidance, within the centre of the BCW, is the COM-B behaviour system. The COM-B system highlights the interaction between capability (C), opportunity (O) and motivation (M) necessary to perform a desired behaviour (34). Behaviour arises as a function of someone’s physical and psychological capability (e.g.: skills and knowledge to perform the behaviour); the physical and social opportunity (e.g.: social cues/norms); and their automatic and reflective motivation (e.g.: impulsive response and cognitive evaluation of the benefit of performing the behaviour) (30, 35). For a behavioural change intervention to be successful, one or more of these three factors in the COM-B system need to change (26, 31, 36).

The layer surrounding the central COM-B system comprises nine intervention functions through which behavioural change is promoted or encouraged (37). Intervention functions are described as *“broad categories by which an intervention can change behaviour”*, emphasising an intervention may have “*more than one function”* [(31) ,p.166]. These functions are linked to the COM-B model, in essence showing more clearly which intervention functions are linked to desired behavioural change, e.g. education intervention affects change in psychological capability and reflective motivation.

The BCW also comprises seven policy categories (37). These policy categories reflect the understanding that sometimes behavioural change occurs due to changes demanded or promoted by relevant authorities which serve to support or enable the adoption of new or revised behaviours (31). For example, improved workplace health and safety practices as a result of legislation introduced by Government on safety and health at work. Lastly, the BCW allows linking of intervention function to behavioural change techniques, which are in essence observable and reproduceable components of the intervention (35). A list of 93 behavioural change techniques have been listed and described for consideration in the behaviour change technique taxonomy to allow for appropriate alignment and operationalisation with intervention functions in the BCW (38).

With this in mind, this study sought to develop an intervention to overcome and remove the psychological barriers to injectable treatment in T2D, within a BCW framework.

## Materials And Methods

### Preliminary Work

The importance of good primary research among those whom the intervention is developed to assist, is key, particularly in patient centric interventions or those targeting behavioural change (22). A systematic approach has been taken to build an evidence base to understand the key psychological barriers to the initiation of, and adherence to, injectables. This approach reflects O’Cathain et al’s framework (39) to support implementation of the Medical Research Council guidance for development and evaluation of complex interventions (25).

A systematic review was undertaken (40) which reported on the need for theory-based, structured diabetes education to focus on associated psychological constructs to inform effective, patient-centric provisions to improve injectable initiation and persistence. More specifically the review found that successful diabetes education relied on facilitating change in participant cognition and behaviours, with psychosocial and behavioural change central in successful interventions. The review also confirmed diabetes education was more effective when led by Health care professionals (HCPs), with peer input, delivered in a group setting (40).

Involving people from a target population in the research development process allows a focus on aspects and experiences that are important for service users; and alignment with patient centric outcomes relevant to the target population (41). To gain such insights, focus groups were conducted with individuals with T2D who had recently commenced injectable therapies. Focus group findings highlighted patient-centric issues, as well as the education requirements, to be addressed to increase uptake and adherence. The four main themes identified within the data were: *1. Beliefs about diabetes and injectable treatments. 2. Knowledge of diabetes and injectables. 3. Barriers to initiation and adherence. 4. Informing education design* (**Supplementary Information: Table of Results**).

Findings from the systematic review and focus groups provided an evidence base to inform development of an intervention for people with T2D transitioning to injectable therapies: Overcoming and Removing Barriers to Injectable Treatment in T2D (ORBIT). The BCW, which captures the range of psychosocial and physical mechanisms necessary for optimal behavioural change, was used to provide a theoretical framework for the development of this intervention. The key stages in the design of interventions can be separated into three key aspects: i) Understanding the behaviour; ii) Identifying intervention options and iii) Identifying content and implementation options (30).

### Understanding the Behaviour

In this phase the foundations for successful intervention are laid, with each subsequent phase building on this initial phase. To address this, and in line with the central system in the BCW, the problem behaviour must be defined, then a target behaviour must be selected and specified with identification of the change(s) required (35, 42).

*Define the problem in behavioural terms:* This step requires definition of the problem, specifying the target group and the behaviour. In developing this intervention, identified gaps in the literature around addressing barriers to uptake and adherence to injectables, coupled with calls to provide interventions to tackle this issue, informed this definition. Therefore, suboptimal uptake and adherence to injectable therapies among T2Ds was defined as the problem to be addressed.

*Select target behaviour:* Although treatment targets exist for HbA_1c_ and related physiological measures, the same consistency is not available for psychological and behavioural aspects. Accordingly, when developing a list of target behaviours for consideration, each should be considered in terms of impact, ease of change and measurement. For this intervention the targeted behaviour was increased uptake of injectable therapies and improved adherence.

*Specify target behaviour:* This aspect requires greater consideration of the target behaviour i.e. Who will perform this behaviour? What do they need to do differently? When will they do this? Where? With whom? How? These questions were addressed in relation to the existing literature and the views gathered from T2Ds using injectables.

• *Behaviour targeted for change:* Increased uptake of injectable therapies and improved adherence
• *When and where is the behaviour performed:* Injections to manage T2D are administered by people with T2D in their home within daily routines, in line with prescribed guidance

*Identify what needs to change:* From the literature, systematic review (40) and focus group findings there are clear barriers and behaviours which need to be changed to address suboptimal uptake and adherence and improve same. These fall into key domains: Patient beliefs/perceptions, side effects, healthcare professional inertia, patient knowledge, psychological aspects, self-efficacy/mastery, control, daily life, education requirements and support (See **Tables 1**, **2**).

**Table 1:** Mapping barriers to initiation/adherence of injectable treatments.

Barriers	Details of Barriers	COM-B Component	Intervention Function Selected
**Patient beliefs/perceptions**	Patients with T2D have misconceptions about injectable therapies, particularly in the absence of symptomology	Psychological Capability	Education
**Side effects**	Patient perceptions of side effects of injectable treatment and associated fear of injections	Reflective Motivation	Education, Persuasion
**HCP inertia**	Patient views of the need to commence injectables affected by clinician inertia	Social Opportunity	Modelling, Enablement
**Patient Knowledge**	Patients have a lack of information of on T2D, treatment targets, and the benefits of injectable therapies from HCPs	1. Psychological Capability 2. Reflective Motivation	1. Education, Training 2. Education, Persuasion, Coercion
**Psychological aspects**	Patients report related issues with aspects such as anxiety and depression	1. Physical Capability 2. Psychological Capability	1. Training, Enablement 2. Education, Training
**Self-efficacy/mastery**	Patient issues exist around their confidence to inject, to effectively use injectable therapies	1. Physical Capability 2. Social Opportunity	1. Training, Enablement 2. Modelling
**Control**	Many patients suggest they feel they cannot control their diabetes	Reflective Motivation	Education, Persuasion
**Daily life**	Patients report issues with injectable treatments and how they ‘fit’ within daily routines	1. Social Opportunity 2. Psychological Capability	1. Modelling, Enablement 2. Education, Training
**Education required**	There is a call for accessible information delivered in a supportive environment	1. Physical Capability 2. Psychological Capability	1. Training, Enablement 2. Education, Training
**Support**	Family Involvement and peer delivery important aspects in any education provision for this groups	Automatic Motivation	Persuasion, Enablement

**Table 2:** Selected behavioural change techniques: translation to intervention.

Barriers	Intervention Function	Behavioural Change Technique	Translation into the Intervention
**Patient beliefs**	Education	5.1: Information about health consequences 13.3: Incompatible beliefs	Provide information on clinical guidance on the need to control T2D Provide information on the progression of T2D Provide information on improved health among those who adhere to injectable treatments Discuss efficacy of injectables and improvements in design
**Side effects**	Education, Persuasion	9.2: Pros and cons 1.2:Problem solving 5.1: Information about health consequences	Compare the advantages and disadvantages of injectables Identify and discuss patient concerns Use clinical guidelines to illustrate causes, symptoms and preventative practices for hypoglycaemia
**HCP inertia**	Modelling, Enablement	9.1.: Credible source	Provide information on guidelines for use of injectable therapies Delivery from HCP advocating importance of initiation and adherence
**Patient Knowledge**	Education, Training, Persuasion, Coercion	5.1: Information about health consequences 7.8: Associative learning	Provide best practice information on treatment targets and benefits of injectable therapies Present visual aids to encourage adoption of healthier behaviours including medication adherence, improved diet and increased activity
**Psychological aspects**	Training, Enablement, Education	5.6: Information about emotional consequences 11.2: Reduce negative emotions 13.2: Framing/Reframing	Explanation that improved glycaemic control can improve quality of life Advise on the use of appropriate techniques to reduce diabetes-related distress Explore the positives of controlling diabetes, rather than the negatives associated with poor control
**Self-efficacy/mastery**	1. Training, Enablement, Modelling	6.1: Demonstration of the behaviour 8.1: Behavioural practice	Demonstration of injectable technique and discussion of injection sites Opportunity for patients to practice injection technique
**Control**	Education, Persuasion	1.4 Action planning 12.3: Avoidance/Reducing exposure to cues for behaviour	Development of action plan to improve self-management and adherence Discussion and consideration of problem behaviours which affect perception of control
**Daily life**	Modelling, Enablement, Education, Training	1.2: Problem solving 1.4 Action planning 6.2: Social comparisons	Work with patients to identify impact of injectables on daily life Patient developed strategies for improved T2D control Show how injectables can be incorporated into the busiest routines
**Education required**	Training, Enablement, Education	5.1: Information about health consequences 15.1: Verbal persuasion about capability 6.3: Information on the approval of others	Information to be delivered *via* a range of media Reinforcement and reassurance offered on patient ability to manage and persevere with injectables Time for peer support and discussion
**Support**	Persuasion, Enablement	6.3: Information on the approval of others	Involve family in the session and discussions to provide support and ensure understanding of injectable therapies

### Identifying Intervention Functions

This stage of the design requires identification and selection of appropriate intervention functions and policy categories. The intervention functions are broad descriptors of methods by which the proposed intervention can serve to change behaviour in the target group (35). Policy categories are external aspects which may help to support intervention delivery.

*Identify intervention functions:* Guided by the COM-B system, 6 of the 9 intervention functions to address problematic behaviour (barriers) were identified: Education, Persuasion, Modelling, Enablement, Training, and Coercion (See **Table 1**).

*Identify policy categories:* When exploring policy categories, it was evident that whilst the majority of the categories could be described as relevant to the intervention design, there were 4 key policy categories that best aligned with the selected intervention functions: guidelines, regulation, legislation and service provision. Guidelines and service provision were deemed to be most appropriate in relation to this intervention.

### Identifying Content and Implementation Options

In the final phase, behavioural change techniques need to be considered alongside a method of delivery for the proposed intervention.

*Identify behaviour change techniques:* Behavioural change techniques were identified using the behaviour change technique taxonomy (38). The techniques were considered in conjunction with the identified barriers, intervention functions and policy categories. The capacity of the behaviour change technique to facilitate change and the potential for translation into the intervention were also used to guide selection (See **Table 2**).

*Identify the mode of delivery:* The mode of delivery is key to the effective translation of the behavioural change techniques into intervention content. The grouping of people with diabetes, their needs and the barriers to injectable initiation and continuation influenced the delivery mode, with face-to-face delivery in small groups identified as preferrable.

## Results

Key guidance and methodology from successful interventions have been used to inform the development of intervention functions, policy categories and behavioural change techniques to overcome and remove barriers to injectable treatment in T2D (31, 34, 43). For this intervention the targeted behaviour was increased uptake of injectable therapies and improved adherence. Key barriers to be overcome in order to improve uptake of and adherence to injectable treatment in those with T2D have been examined within the existing literature and also captured *via* discussions with respondents with T2D currently prescribed injectable therapies. The identified barriers have been used to inform the intervention, within the BCW Framework, with these mapped onto the relevant COM-B components, intervention functions and selected behavioural change techniques (**Table 1**).

The final phase in this systematic process called for the identification of content and behaviour change techniques. **Table 2** describes the behavioural change techniques drawn together from the COM-B system and intervention functions outlined in **Table 1**. In line with feedback from patients and guided by the literature, the newly developed ORBIT intervention consists of the delivery of the identified behavioural change techniques to people with diabetes prior to commencing injectable treatment.

## Discussion

The systematic process within the BCW framework provides a clear pathway for intervention design. The BCW has been used to develop interventions to facilitate a variety of behaviour change, in various contexts and across a range of populations (e.g. 34,35,43). At the centre of the model sits a deceptively simple behavioural system that takes cognisance of the key aspects required to engage in a given behaviour (32). The COM-B system allowed for clear identification of the behaviour targeted for change, but also provided a basis for the selection of intervention functions. It is important that as the researcher moves through the related steps in the BCW they do not become overwhelmed by the number of options or the variety of behavioural change techniques. Key to this clarity is the preparatory work or primary research that is essential to inform intervention design when using the BCW (22). This preparatory work is essential to ensure that patient-centric interventions, such as ORBIT, are developed in line with the needs of people with diabetes rather than relying on *“practitioner or researcher intuition”* (23,p.1). The use of the BCW framework in this process has ensured the development of the intervention is theory driven, with the research able to be more readily validated through replication due to the clarity around processes and tasks completed at each stage. The BCW responds to the call from the MRC for the use of theoretical frameworks in the development of behavioural change interventions to ensure accurate identification of the mechanisms for behavioural change (25). It appears the BCW provides a simple and systematic framework for intervention design to affect behavioural change.

As noted in the *Results*, the newly developed ORBIT intervention will allow for delivery of the identified behavioural change techniques to people with diabetes prior to commencing injectable treatment. In line with the translation of the behavioural change techniques, best practice guidelines and clinical guidelines will be used to guide same (20, 33, 40). Responding to this, the intervention should be delivered in a group setting, led by a trained HCP (Diabetes Specialist Dietitian), with opportunity for peer discussion and support. Participants should also receive related support materials. This intervention was designed to overcome and remove the psychological barriers to injectable treatment in T2D, to improve uptake and adherence to injectables. As such ORBIT has the capacity to improve outcomes for those living with T2D through better glycaemic control, by hastening adoption of injectables after prescription and helping ensure continued use.

### Limitations

Simply because the framework is systematic and well-devised does not ensure its effectiveness. This is dependent on the accurate operationalising of behaviours, identification of barriers, intervention categories and behavioural change techniques. However, even then success is not guaranteed as the skill is in the translation of behavioural change techniques into intervention content to ensure the intervention addresses the target behaviour within the grouping of people with diabetes. In this instance, although the intervention has been devised and the content developed, it needs to be evaluated with respect to the removal of the barriers it has been designed to overcome. In essence, the intervention should reduce or remove the identified barriers to injectable initiation and treatment adherence.

### Directions for Future Research

Trialling of the ORBIT intervention would be required. Appropriate reliable and valid individual reported outcome measures of identified barriers. The literature in the area and findings from the systematic review suggest these measures may assess people’s perceptions of their condition or condition related distress, knowledge, self-efficacy/mastery, anxiety, depression, and control should be utilised prior to, and following this intervention to evaluate efficacy. It should not be forgotten that the reduction in levels of depression, anxiety and distress, alongside improvements in knowledge, self-efficacy/mastery and control will not only evidence intervention efficacy; but should ultimately serve to improve injectable use and treatment persistence. To more objectively assess this, it would be important to monitor medication usage, weight (BMI) and HbA_1c_ among those taking part in the intervention to add support for changes in the reported outcome measures by people with diabetes.

## Data Availability Statement

The raw data supporting the conclusions of this article will be made available by the authors, without undue reservation.

## Ethics Statement

Ethical approval was granted by the Office of Research Ethics Committee in Northern Ireland, with governance from the Trust Research Governance Committee prior to the commencement of participant recruitment (15/NI/0091). All participants provided informed consent. The patients/participants provided their written informed consent to participate in this study.

## Author Contributions

DM, AH and VC designed the study. DM, AH, VC, KM, and MO’K were active in the development of the preliminary work. KM and DM guided the development of the intervention via the BCW methodology. KM and DM wrote the first draft of the manuscript. AH and VC critically advised on important intellectual content and contributed to revising of the manuscript. All authors read and approved the manuscript for submission.

## Funding

This work was supported by the HSC Research and Development Division of the Public Health Agency, Northern Ireland (grant number: EAT/4909/13).

## Conflict of Interest

The authors declare that the research was conducted in the absence of any commercial or financial relationships that could be construed as a potential conflict of interest.

## Publisher’s Note

All claims expressed in this article are solely those of the authors and do not necessarily represent those of their affiliated organizations, or those of the publisher, the editors and the reviewers. Any product that may be evaluated in this article, or claim that may be made by its manufacturer, is not guaranteed or endorsed by the publisher.

## References

[1] Zullig LL Gellad WF Moaddeb J Crowley MJ Shrank W Granger BB. Improving Diabetes Medication Adherence: Successful, Scalable Interventions. Patient Prefer Adherence (2015) 9:139–49. doi: 10.2147/PPA.S69651 PMC4315534 25670885

[2] Gogas Yavuz D Ozcan S Deyneli O. Adherence to Insulin Treatment in Insulin-Naïve Type 2 Diabetic Patients Initiated on Different Insulin Regimens. Patient Prefer Adherence (2015) 9:1225–31. doi: 10.2147/PPA.S87935 PMC4556254 26346988

[3] Peyrot M Barnett AH Meneghini LF Schumm-Draeger PM. Insulin Adherence Behaviours and Barriers in the Multinational Global Attitudes of Patients and Physicians in Insulin Therapy Study. Diabetes Med (2012) 29(5):682–9. doi: 10.1111/j.1464-5491.2012.03605.x PMC3433794 22313123

[4] Perez-Nieves M Kabul S Desai U Ivanova JI Kirson NY Cummings AK. Basal Insulin Persistence, Associated Factors, and Outcomes After Treatment Initiation Among People With Type 2 Diabetes Mellitus in the US. Curr Med Res Opin (2016) 32(4):669–80. doi: 10.1185/03007995.2015.1135789 26703951

[5] Perez-Nieves M Ivanova JI Hadjiyianni I Zhao C Cao D Schmerold L. Basal Insulin Initiation Use and Experience Among People With Type 2 Diabetes Mellitus With Different Patterns of Persistence: Results From a Multi-National Survey. Curr. Med. Res. Opin (2017) 33(10):1833–42. doi: 10.1080/03007995.2017.1341403 28604111

[6] Edelman S Pettus J. Challenges Associated With Insulin Therapy in Type 2 Diabetes Mellitus. Am J Med (2014) 127(10 Suppl):S11–6. doi: 10.1016/j.amjmed.2014.07.003 25282008

[7] Polonsky WH Fisher L Guzman S Villa-Caballero L Edelman SV. Psychological Insulin Resistance in Patients With Type 2 Diabetes: The Scope of the Problem. Diabetes Care (2005) 28(10):2543–5. doi: 10.2337/diacare.28.10.2543 16186296

[8] Marrero DG. Overcoming Patient Barriers to Initiating Insulin Therapy in Type 2 Diabetes Mellitus. Clin Cornerstone (2007) 8(2):33–43. doi: 10.1016/s1098-3597(09)60006-5 18357954

[9] Aghili R Ridderstråle M Farshchi A Valojerdi AE Banazadeh Z Malek M. Psychosocial Factors and Glycemic Control in Insulin-Naïve and Insulin-Experienced People With Type 2 Diabetes: A Path Analysis Model. Int. J Diabetes Dev Ctries (2018) 38:289–97. doi: 10.1007/s13410-017-0581-2

[10] Nefs G Pop VJ Denollet J Pouwer F. The Longitudinal Association Between Depressive Symptoms and Initiation of Insulin Therapy in People With Type 2 Diabetes in Primary Care. PloS One (2013) 8(11):e78865. doi: 10.1371/journal.pone.0078865 24223860 PMC3815321

[11] Lee KP. Psycholosocial Factors Associated With Psychological Insulin Resistance in Primary Care Patients in Hong Kong. J Clin Transl Endocrinol (2015) 2(4):157–62. doi: 10.1016/j.jcte.2015.10.001 PMC5685026 29159120

[12] McGuigan K Hill A Coates V O'Kane M Thompson DR Ski CF. Moderating the Relationship Between Diabetes Distress and Mastery: The Role of Depression and Empowerment. Psychol Health Med (2021) 1:1–10. doi: 10.1080/13548506.2021.1894343 33641545

[13] Peyrot M Rubin RR Kruger DF Travis LB. Correlates of Insulin Injection Omission. Diabetes Care (2010) 33(2):240–5. doi: 10.2337/dc09-1348 PMC2809256 20103556

[14] Gonzalez JS Tanenbaum ML Commissariat PV. Psychosocial Factors in Medication Adherence and Diabetes Self-Management: Implications for Research and Practice. Am Psychol (2016) 71(7):539–51. doi: 10.1037/a0040388 PMC5792162 27690483

[15] Moss-Morris R Weinman J Petrie K Horne R Cameron L Buick D. The Revised Illness Perception Questionnaire (IPQ-R). Psychol Health (2002) 17(1):1–16. doi: 10.1080/08870440290001494

[16] Hudson JL Bundy C Coventry PA Dickens C. Exploring the Relationship Between Cognitive Illness Representations and Poor Emotional Health and Their Combined Association With Diabetes Self-Care. A Systematic Review With Meta-Analysis. J Psychosom Res (2014) 76(4):265–74. doi: 10.1016/j.jpsychores.2014.02.004 24630175

[17] Allen NA Zagarins SE Feinberg RG Welch G. Treating Psychological Insulin Resistance in Type 2 Diabetes. J Clin Transl Endocrinol (2016) 7:1–6. doi: 10.1016/j.jcte.2016.11.005 29067243 PMC5651283

[18] Sorli C Heile MK. Identifying and Meeting the Challenges of Insulin Therapy in Type 2 Diabetes. J Multidiscip Healthc (2014) 7:267–82. doi: 10.2147/JMDH.S64084 PMC4086769 25061317

[19] McSharry J Byrne M Casey B Dinneen SF Fredrix M Hynes L. Behaviour Change in Diabetes: Behavioural Science Advancements to Support the Use of Theory. Diabetes Med (2020) 37(3):455–63. doi: 10.1111/dme.14198 31797455

[20] National Institute for Health and Care Excellence. Diabetes in Adults. NICE Quality Standard [QS6]. London: NICE (2016).

[21] Weinger K Beverly EA. Barriers to Achieving Glycemic Targets: Who Omits Insulin and Why? Diabetes Care (2010) 33(2):450–2. doi: 10.2337/dc09-2132 PMC2809300 20103561

[22] Owens C Farrand P Darvill R Emmens T Hewis E Aitken P. Involving Service Users in Intervention Design: A Participatory Approach to Developing a Text-Messaging Intervention to Reduce Repetition of Self-Harm. Health Expect (2011) 14(3):285–95. doi: 10.1111/j.1369-7625.2010.00623.x PMC5060584 20860777

[23] Cane J O'Connor D Michie S. Validation of the Theoretical Domains Framework for Use in Behaviour Change and Implementation Research. Implement Sci (2012) 7:37. doi: 10.1186/1748-5908-7-37 22530986 PMC3483008

[24] Michie S van Stralen MM West R. The Behaviour Change Wheel: A New Method for Characterising and Designing Behaviour Change Interventions. Implement Sci (2011) 6:42. doi: 10.1186/1748-5908-6-42 21513547 PMC3096582

[25] Craig P Dieppe P Macintyre S Michie S Nazareth I Petticrew M. Medical Research Council Guidance. Developing and Evaluating Complex Interventions: The New Medical Research Council Guidance. BMJ (2008) 337:a1655. doi: 10.1136/bmj.a1655 18824488 PMC2769032

[26] Webb J Foster J Poulter E. Increasing the Frequency of Physical Activity Very Brief Advice for Cancer Patients. Development of an Intervention Using the Behaviour Change Wheel. Public Health (2016) 133:45–56. doi: 10.1016/j.puhe.2015.12.009 26822162

[27] Abraham C Kelly MP West R Michie S. The UK National Institute for Health and Clinical Excellence Public Health Guidance on Behaviour Change: A Brief Introduction. Psychol Health Med (2009) 14(1):1–8. doi: 10.1080/13548500802537903 19085307

[28] Roter DL Hall JA Merisca R Nordstrom B Cretin D Svarstad B. Effectiveness of Interventions to Improve Patient Compliance: A Meta-Analysis. Med Care (1998) 36(8):1138–61. doi: 10.1097/00005650-199808000-00004 9708588

[29] Norris SL Engelgau MM Narayan KM. Effectiveness of Self-Management Training in Type 2 Diabetes: A Systematic Review of Randomized Controlled Trials. Diabetes Care (2001) 24(3):561–87. doi: 10.2337/diacare.24.3.561 11289485

[30] Atkins L Michie S. Designing Interventions to Change Eating Behaviours. Proc Nutr Soc (2015) 74(2):164–70. doi: 10.1017/S0029665115000075 25998679

[31] Lefevre CE. The Behavioural Change Wheel in Action: Applying Behaviour Change Methods and Techniques to Environmental Issues. London: Centre for Behaviour Change, UCL (2016).

[32] Michie S Atkins L West R. The Behaviour Change Wheel: A Guide to Designing Interventions. Great Britain: Silverback Publishing (2014). 322 p.

[33] National Institute for Health and Care Excellence. Behaviour Change Individual Approaches. NICE Public Health Guideline [PH49]. London: NICE (2014).

[34] Barker F Atkins L de Lusignan S. Applying the COM-B Behaviour Model and Behaviour Change Wheel to Develop an Intervention to Improve Hearing-Aid Use in Adult Auditory Rehabilitation. Int J Audiol (2016) 55(Suppl 3):S90–8. doi: 10.3109/14992027.2015.1120894 27420547

[35] Mc Sharry J Murphy PJ Byrne M. Implementing International Sexual Counselling Guidelines in Hospital Cardiac Rehabilitation: Development of the CHARMS Intervention Using the Behaviour Change Wheel. Implement Sci (2016) 11(1):134. doi: 10.1186/s13012-016-0493-4 27724957 PMC5057276

[36] Smith CM Griffiths F Fothergill RT Vlaev I Perkins GD. Identifying and Overcoming Barriers to Automated External Defibrillator Use by GoodSAM Volunteer First Responders in Out-of-Hospital Cardiac Arrest Using the Theoretical Domains Framework and Behaviour Change Wheel: A Qualitative Study. BMJ Open (2020) 10(3):e034908. doi: 10.1136/bmjopen-2019-034908 PMC7066637 32161161

[37] Carney R Bradshaw T Yung AR. Physical Health Promotion for Young People at Ultra-High Risk for Psychosis: An Application of the COM-B Model and Behaviour-Change Wheel. Int J Ment Health Nurs (2016) 25(6):536–45. doi: 10.1111/inm.12243 PMC6853191 27432534

[38] Michie S Richardson M Johnston M Abraham C Francis J Hardeman W. The Behavior Change Technique Taxonomy (V1) of 93 Hierarchically Clustered Techniques: Building an International Consensus for the Reporting of Behavior Change Interventions. Ann Behav Med (2013) 46(1):81–95. doi: 10.1007/s12160-013-9486-6 23512568

[39] O'Cathain A Croot L Duncan E Rousseau N Sworn K Turner KM. Guidance on How to Develop Complex Interventions to Improve Health and Healthcare. BMJ Open (2019) 9(8):e029954. doi: 10.1136/bmjopen-2019-029954 PMC6701588 31420394

[40] McCay D Hill A Coates V O'Kane M McGuigan K. Structured Diabetes Education Outcomes: Looking Beyond HbA1c. A Systematic Review. Pract. Diabetes (2019) 36:86–90. doi: 10.1002/pdi.2221

[41] Public Health Agency. HSC Research and Development Division: Strategy for Personal and Public Involvement in Health and Social Care Research. Belfast: PHA (2010).

[42] Sargent L McCullough A Del Mar C Lowe J. Using Theory to Explore Facilitators and Barriers to Delayed Prescribing in Australia: A Qualitative Study Using the Theoretical Domains Framework and the Behaviour Change Wheel. BMC Fam Pract (2017) 18(1):20. doi: 10.1186/s12875-017-0589-1 28193174 PMC5307801

[43] Suntornsut P Wongsuwan N Malasit M Kitphati R Michie S Peacock SJ. Barriers and Recommended Interventions to Prevent Melioidosis in Northeast Thailand: A Focus Group Study Using the Behaviour Change Wheel. PloS Negl Trop Dis (2016) 10(7):e0004823. doi: 10.1371/journal.pntd.0004823 27472421 PMC4966968

