# Supplementary material for: Overcoming Barriers to Injectable Therapies: Development of the ORBIT Intervention Within a Behavioural Change Framework
Source: Front Clin Diabetes Healthc. 2021 Dec 9;2:792634. doi: 10.3389/fcdhc.2021.792634 (PMC10012154; doi:10.3389/fcdhc.2021.792634)
Supplement: Supplementary file 1 [file DataSheet_1.docx]

**SUPPLEMENTARY MATERIALS**

**Focus groups**

Injectable therapies can offer a high level of efficacy for glycaemic control for those with Type 2 Diabetes (T2D). However, the transition to injections remains a problematic one. Patient attitudes, beliefs and knowledge around injectable use are key predictors for initiation of injectable therapies and for continued adherence. To inform intervention development, focus groups were conducted to understand the experiences of those with T2D who have recently transitioned to injectable therapies, to identify issues/barriers associated with initiation and medication adherence; and inform education provision among this group.

To avoid the use of preconceived or predicted categories, an inductive approach was used to ensure themes and categories emerged unrestricted from the data, i.e.: the data generates the explanation (1). Accordingly, conventional analysis was used to effectively capture and describe all aspects of participant experience (2).

Participants, within a local health trust in Northern Ireland, were identified using the diabetes management system within the Trust. The register holds data on individuals diagnosed with diabetes, including information about their treatment regime. All met the inclusion criteria:

- Diagnosed with T2D for 1 year or more
- Aged between 25-75 years
- Commenced insulin or GLP-1receptor agonists within the previous 12 months

Purposeful selection of potential participants (differing locations, age groups, gender, injectable regimens) was used to allow for a range of experiences on the use of injectables to be reflected. Across the Trust, a total of 36 potential participants were identified. Potential participants were invited to attend focus groups to discuss their experiences of starting injectable treatment and how that experience could be improved. It was hoped that between 8-12 participants would attend each group. Of the 36 identified, 8 were unable to be contacted (22.2%). Of the remaining 28; 8 declined the invitation (22.2%), a further 8 did not attend the focus group (22.2%) and 1 had passed away (2.8%). Eleven participants (9 males; 2 females) attended the focus groups. Participants were facilitated across 3 separate groups.

All groups were guided by a semi-structured topic guide (Table 1), facilitated by a specialist diabetes dietitian and audio recorded, with participant consent, to allow for verbatim transcription and analysis of the resulting information. The qualitative data was stored and managed in NVivo, (v.11) (3). In line with the inductive approach, conventional content analysis was used to label, code and categorise the data to elucidate common themes and accurately describe participant experience (4). Analysis was ceased when saturation was reached, i.e.: no further new themes or categories were found (5). Internal peer review of the themes was carried out by a specialist dietician and a psychologist. Following internal peer review, ongoing consultation among two diabetes specialist dietitians, a psychologist and two diabetes healthcare specialists involved the questioning, clarification, operationalising and refining of all themes and associated categories. This process was recorded throughout (audit trail).

Eleven participants with T2D (9 males; 2 females) aged 47 to 74 years (M=60, SD=9.3) participated in the focus groups. All participants were treated using injectable therapies (4 Insulin only; 4 GLP-1 only; 3 Insulin and GLP-1 mix). Four main themes were identified within the data: *1. Beliefs about diabetes and injectable treatments; 2. Knowledge of diabetes and injectables; 3. Barriers to initiation and adherence; 4. Informing education design.*

**References**

1. Fade SA, Swift JA. Qualitative research in nutrition and dietetics: data analysis issues. *J Human Nutr Diet* (2010) 24:106-114
2. Sandelowski M. Whatever happened to qualitative description? *Res Nurs Health* (2000) 23:334-340
3. QSR International Pty Ltd. NVivo Qualitative Data Analysis Software: Version 11. (2016)
4. Hsieh H-F, Shannon SE. Three approaches to qualitative content analysis*. Qual Health Res* (2005) 15:1277-1288
5. Saunders B, Sim J, Kingstone T, et al. Saturation in qualitative research: exploring its conceptualization and operationalization. *Qual Quant* (2018) 52:1893-1907

**SUPPLEMENTARY MATERIALS**

**Table 1: Topic Guide**

| **Topic Guide** |
| --- |
| 1. Think back to when you were first told that you needed injections. Did it cause you any concern? |
| 1. Did anyone in the group experience stress at the thought of injections? |
| 1. Was there anything in particular that was putting you off going on an injection? |
| 1. Most of you will have gone through a process of being on tablets only for several years before starting injections. At what point do you feel we should have started preparing you for injection treatment? |
| 1. If we told you that we had a group programme to attend to prepare you for injections would you have found this useful and what would be useful to include in such a programme? |
| 1. I asked you earlier about stress. In there anything specific that we could include in the programme that would help reduce this? |
| 1. How would you like the education to be delivered? |
| 1. What written information would you like to take away with you at the end of the sessions to support you at home? |
| 1. It’s planned to have 2 sessions in the programme lasting 1.5 to 2hrs each. Would you be happy with this? |
| 1. Is there anything else that you would like to share that you think is important or useful? |

**SUPPLEMENTARY MATERIALS**

**Table 2: Table of Results: Findings from Focus Groups**

| **Key Themes** | **Categories** |
| --- | --- |
| 1. **Beliefs about Diabetes/ Injectable Treatments** | 1.1: Diabetes is a less serious condition  1.2: Progression of treatment options  1.3: Perceptions about needles  1.4: Diabetes is in control of me |
| **2.** **Knowledge** | 2.1: Lack of knowledge around condition  2.2: Lack of knowledge around hypos and optimal insulin levels  2.3: Lack of timely information  2.4: Family history |
| **3.** **Barriers** | 3.1: Shame, Disappointment, Blame  3.3: Injecting – Fear, pain, bruising  3.4: Need to drive/work  3.5: Weight gain  3.6: Complexity of medication |
| **4.** **Informing Education Design** | 4.1: Group education with peers  4.2: Family education  4.3: One-off session  4.4: Early intervention  4.5: Involve, don’t ‘talk at’  4.6: Deal with psychological aspects |
